# Data on deviance predictability in the assessment of mismatch negativity in patients with schizophrenia

**DOI:** 10.1016/j.dib.2016.03.045

**Published:** 2016-03-17

**Authors:** Magdalena Horacek, Christian Kärgel, Norbert Scherbaum, Bernhard W. Müller

**Affiliations:** a Department of Psychiatry and Psychotherapy, LVR-Hospital Essen, Faculty of Medicine, University of Duisburg-Essen, Virchowstrasse 174, 45147 Essen, Germany; b Department of Psychology, University of Wuppertal, Gaußstrasse 2 0, 42119 Wuppertal, Germany; c Department of Addiction Medicine and Addictive Behavior, LVR-Hospital Essen, Faculty of Medicine, University of Duisburg-Essen, Virchowstrasse 174, 45147 Essen, Germany

**Keywords:** Cognitive Neuroscience, Neurophysiology,, Mismatch Negativity

## Abstract

We investigated the MMN at electrode Fz to 12% temporally predictable or unpredictable duration decrement deviant stimuli in 29 healthy controls and 31 schizophrenia patients. With a stimulus onset asynchronicity of 500 ms in the regular predictable condition, a deviant occurred every 4 s while it varied randomly in the unpredictable condition.

Here we report detailed data tables and multivariate analysis of variance results (MANOVA) on MMN, P3a and standard ERP data including details on follow-up analyses. An extended figure shows MMN difference curves and averages to standard and deviant stimuli in both experimental conditions and subject groups.

**Specifications table**

**Table:** 

Subject area	*Cognitive Neuroscience*
More specific subject area	*Neurophysiology, Mismatch Negativity (MMN)*
Type of data	*Tables, Figure*
How data was acquired	*Electroencephalography (EEG), evoked potentials (ERPs)*
Data format	*Processed*
Experimental factors	*Two groups (schizophrenia patients, controls), temporal predictability of deviant stimuli (random/fixed)*
Experimental features	*Assessment of MMN, P3a and standard ERPs to 12% duration decrement deviant stimuli in 29 healthy controls and 31 schizophrenia patients. With a stimulus onset asynchronicity of 500 ms a deviant occurred every 4 seconds in the predictable condition while it varied randomly in the unpredictable condition.*
Data source location	*Essen, Germany*
Data accessibility	*Data are provided with this article*

## **Value of the data**

• We report data on statistical analyses which may be of interest for other researchers when i.e. aiming to calculate sample sizes for future studies in this field of research.
• We show an extended figure demonstrating how the mismatch negativity is generated by evoked response potential curves to standard and to deviant stimuli in the predictable and unpredictable deviance conditions.
• We report data on ERP components to standard and deviant stimuli in patients and in controls which are not part of the MMN but which may be of specific interest for those doing evoked potential research in schizophrenia patients or other clinical samples.

## 1 Data

**Fig. 1:**
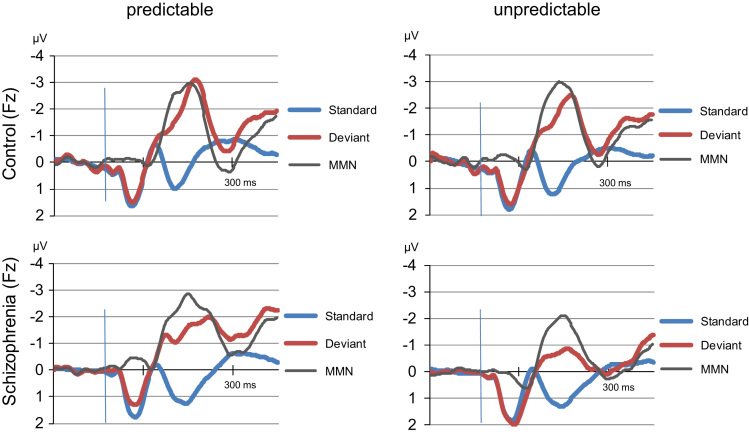
Grand mean evoked response potential curves to standard and deviant stimuli and mismatch negativity (MMN) difference waves in two conditions (predictable & unpredictable) and two groups (patients & controls) at electrode Fz (µV).

The data give details of analyses of the dependent variable mismatch negativity (MMN) and other evoked response potential (ERP) components in relation to the experimental factors. We assessed two groups (patients with schizophrenia and controls) and used two experimental conditions (with and without predictability of the deviant stimulus). Fig. 1 shows grand mean ERP curves to standard and deviant stimuli and the resulting MMN difference wave in both groups and both conditions (Table 1, Table 2, Table 3, Table 4, Table 5, Table 6, Table 7, Table 8, Table 9, Table 10, Table 11, Table 12).

**Table 1:** MANOVA MMN mean amplitude 2 group (between factor patient/control)×2 predictability (within factor fixed/random) analysis of variance.

	*F*	Df	Sig
Main effect group	1.651	1/58	.204
Main effect predictability	4.797	1/58	.033
Interaction group×predictability	4.087	1/58	.048

**Table 2:** *T*-tests on the effect of group (patient/control) on MMN amplitudes in (a) in the predictable condition and (b) in the unpredictable condition.

	*T*	Df	Sig
(a) Predictable group	1.51	58	.880
(b) Unpredictable group	−2.32	58	.023

**Table 3:** MMN mean amplitudes in a predictability (within factor: fixed/random) analysis of variance (a) in controls and (b) in patients with schizophrenia.

	*F*	Df	Sig
(a) Within effect predictability controls	0.018	1/28	.894
(b) Within effect predictability patients	7.538	1/30	.010

**Table 4:** Pearson correlation between **control subjects** general and cognition variable and mean MMN amplitudes in fixed and random condition and fixed-random difference score (*p*<.01 in bold).

Pearson correlation	MMN mean amplitude fixed (*r*, sign.)	MMN mean amplitude random (*r*, sig)	MMN mean amplitude random−fixed difference score (*r*, sig)
**Control subjects**			
Age (yrs)	.1.6 (*p*=.39)	−.29 (*p*=.11)	−.39 (*p*=.03)
Education (yrs)	−.05 (*p*=.67)	−.28 (*p*=.13)	−.19 (*p*=.13)
MWT-B (IQ score)	−.10 (*p*=.58)	−.40 (*p*=.02)	−.24 (*p*=.21)

**Table 5:** Pearson correlation between **schizophrenia patients** general, clinical and cognition variables and mean MMN amplitudes in fixed and random condition and fixed-random difference score.

Pearson correlation	**MMN mean amplitude fixed** (*r*, sig)	**MMN mean amplitude random** (*r*, sig)	**MMN mean amplitude random−fixed difference score** (*r*, sig)
**Schizophrenia patients**			
Age (yrs)	.17 (*p*=.34)	−.01 (*p*=.92)	−.16 (*p*=.37)
Education (yrs)	.05 (*p*=.76)	−.25 (*p*=.17)	−.25 (*p*=.17)
MWT-B (IQ score)	.31 (*p*=.08)	.11 (*p*=.52)	−.17 (*p*=.34)
**Clinical**			
Schizophrenia onset (age)	.12 (*p*=.52)	−.10 (*p*=.57)	−.18 (*p*=.31)
Schizophrenia duration (yrs)	.12 (*p*=.51)	.07 (*p*=.67)	−.04 (*p*=.82)
PANSS positive (score)	−.07 (*p*=.70)	−.03 (*p*=.85)	.03 (*p*=.85)
PANSS negative (score)	.01 (*p*=.97)	.01 (*p*=.92)	.01 (*p*=.96)
PANSS global (score)	−.09 (*p*=.59)	.06 (*p*=.72)	.13 (*p*=.45)
PANSS total (score)	−.06 (*p*=.71)	.02 (*p*=.88)	.07 (*p*=.67)
GAF (score)	−.16 (*p*=.38)	−.32 (*p*=.07)	−.11 (*p*=.52)

**Table 6:** P3a component amplitude data derived from the difference wave (mean, sd [µV]).

P3a Fz amplitudes	Patient [mean (sd) µV]	Control [mean (sd )µV]
Unpredictable	0.41 (1.73)	−0.16 (1.55)
Predictable	−0.38 (1.84)	0.30 (1.27)

**Table 7:** MANOVA on P3a amplitudes: 2 group (between factor patient/control)×2 predictability (within factor fixed/random) multivariate analysis of variance.

	*F*	Df	Sig
Main effect group	0.03	1/58	.875
Main effect predictability	0.43	1/58	.512
**Interaction group**×**predictability**	**6.25**	1/58	**.015**

**Table 8:** MANOVA on P3a amplitudes: within factor predictability (fixed/random) analysis of variance in each group (patient/control) separately.

	*F*	Df	Sig
Effect of predictability controls	2.28	1/28	.142
Effect of predictability patients	4.08	1/30	.052

**Table 9:** *T*-tests P3a amplitudes: effect of group (patient/control) within each condition (predictable/unpredictable).

	*T*	Df	Sig
Predictable	1.67	53.6	.103
Unpredictable	−1.35	58	.182

**Table 10:** P50, N100, and P200 ERP data to standard and deviant stimuli: amplitude (µV) and latency (ms) data.

	**Patient (mean (sd))**	**Control (mean (sd))**
**Amplitudes (µV)**		
STD P50 fixed	2.21 (1.23)	2.03 (1.06)
STD P50 random	2.12 (1.04)	2.17 (0.99)
STD N100 fixed	−0.74 (1.51)	−1.12 (1.48)
STD N100 random	−0.49 (1.26)	−0.85 (1.62)
STD P200 fixed	2.03 (1.39)	1.43 (1.13)
STD P200 random	1.81 (1.13)	1.63 (1.10)
DEV P50 fixed	1.87 (1.60)	1.93 (1.18)
DEV P50 random	2.37 (1.85)	2.04 (1.12)

**Latencies (ms)**		
STD P50 fixed	76.58 (11.03)	74.00 (14.66)
STD P50 random	77.29 (10.26)	76.55 (11.99)
STD N100 fixed	127.81 (17.32)	124.34 (12.73
STD N100 random	129.16 (13.29)	127.93 (14.74)
STD P200 fixed	183.10 (26.60)	172.55 (19.38)
STD P200 random	178.90 (26.32)	174.76 (19.37)
DEV P50 fixed	72.58 (12.43)	73.10 (11.62)
DEV P50 random	78.90 (13.31)	73.31 (15.52)

**Table 11:** MANOVA analyses of ERP components to standard stimuli (STD) and to deviant stimuli (DEV). Group (patient/control)×predictability (random/fixed) analyses.

MANOVA		Main effect group (patient/control)			Main effect predictability (fixed/random)			Interaction group×predictability		
		*F*	Df	Sig	*F*	Df	Sig	*F*	Df	Sig
Amp.	P50 STD	0.05	1/58	.823	0.05	1/58	.819	0.94	1/58	.335
	N100 STD	1.05	1/58	.309	**4.53**	**1**/**58**	**.038**	0.01	1/58	.929
	P200 STD	1.98	1/58	.165	0.01	1/58	.910	2.26	1/58	.139
Lat.	P50 STD	0.34	1/58	.565	1.82	1/58	.182	0.58	1/58	.449
	N100 STD	0.49	1/58	.485	1.90	1/58	.173	0.39	1/58	.536
	P200 STD	2.05	1/58	.157	0.10	1/58	.754	1.03	1/58	.315

**Table 12:** MANOVA analysis on P50 data to standard and deviant stimuli in a group (patient/control)×predictability (fixed/random)×stimulus (P50 to standards, P50 to deviants) analysis.

P50 group×predictability×stimulus	*F*	Df	Sig
Main effect group (patient/control)	0.12	1/58	.734
Main effect stimulus (standard/deviant)	2.14	1/58	.149
Main effect predictability (fixed/random)	0.59	1/58	.446
Interaction group×predictability	0.11	1/58	.739
Interaction predictability×stimulus	1.28	1/58	.263
Interaction stimulus×group	0.13	1/58	.718
Interaction predictability×stimulus×group	1.60	1/58	.212

## 2 Experimental design, materials and methods

We assessed 31 patients with schizophrenia and 29 control subjects. Groups did not differ in years in education, amount of smokers and a proxy of verbal IQ (MWT-B) [5]. Exclusion criteria were age exceeding 18–55 yrs, alcohol or drug abuse or dependency or past dependencies less than 1 year ago, acute neurological or DSM-IV axis-I disorders other than schizophrenia or schizoaffective disorder and current benzodiazepine medication.

### 2.1 Clinical assessments

Diagnoses were verified by means of Structured Clinical Interview for DSM**-**IV (SCID)[7] in patients and short diagnostic interviews for DSM-IV diagnoses in controls (Mini-DIPS) [6]. Clinical symptom assessments comprised the Positive and Negative Syndrome Scale (PANSS) [4] and the Global Assessment of Functioning Scale (GAF) [1].

### 2.2 Stimuli and experimental design

Auditory stimuli presented with a 500 ms stimulus onset asynchrony. Sine-wave tones were 1 kHz, 80 dBA and 80 ms with rise and fall times of 10 ms. Deviant tones had a duration of 40 ms with 5 ms rise/fall time. Deviant probability was 12% in both conditions. In the fixed predictable condition the fourth stimulus was a duration deviant stimulus "D", the other stimuli were standards "S" resulting in a series of "SSSDSSSS" stimuli. In the unpredictable random condition, the duration deviant stimulus occurred randomly at the second to eight position in the train of eight stimuli. No deviants occurred in direct succession. Stimuli were presented in two runs using "Presentation" (V.14.1, Neurobehavioral Systems Inc.) software while participants watched a silent nature film (visual angle 5°).

### 2.3 EEG recording and analysis

EEG was recorded at Fz, Fcz, Cz, C3 and C4. Linked earlobes were used as reference and AFz as ground. EOG was recorded to monitor vertical and horizontal eye movements. EEG was recorded with a band pass filter of 1.5–250 Hz and a digitisation rate of 500 Hz. Trials with artefacts were excluded and data were passed through an IIR Butterworth zero 30 Hz low pass filter (48 dB/oct) [2]. Eye blinks were corrected using a regression method [3]. Segments were computed from 100 ms before to 400 ms after stimulus onset and baseline-corrected. Epochs exceeding ±50 µV were rejected from further analysis. Single subject averages were computed for stimulus types and conditions. MMN difference waves were obtained and peak amplitudes and latencies were determined at electrode site Fz, which showed the largest MMN amplitude in this study. MMN mean amplitudes comprising data points ±50 ms around individual peak amplitudes were computed. P3a was assessed from MMN difference curves for the largest positive deflection following the MMN. Additionally, we measured amplitudes and latencies of standard ERP components P50, N100 and P200 in ERP average waveforms to standard stimuli and the P50 component from ERP average waveforms to deviant stimuli.
